# Bi_2_Te_3_-based applied thermoelectric materials: research advances and new challenges

**DOI:** 10.1093/nsr/nwaa259

**Published:** 2020-10-17

**Authors:** Jun Pei, Bowen Cai, Hua-Lu Zhuang, Jing-Feng Li

**Affiliations:** State Key Laboratory of New Ceramics and Fine Processing, School of Materials Science and Engineering, Tsinghua University, China; State Key Laboratory of New Ceramics and Fine Processing, School of Materials Science and Engineering, Tsinghua University, China; State Key Laboratory of New Ceramics and Fine Processing, School of Materials Science and Engineering, Tsinghua University, China; State Key Laboratory of New Ceramics and Fine Processing, School of Materials Science and Engineering, Tsinghua University, China

Thermoelectric (TE) materials can directly exchange heat and electricity in the solid state. TE devices that do not contain moving parts or produce emissions have been commercialized as electronic coolers and temperature stabilizers, such as refrigerators, wine cellars, portable coolers and temperature controllers for optical communication equipment, infrared sensors and high-powered lasers. TE cooling is suitable for both localized and active cooling and may be the only viable solution to heat management challenges that restrict the development of next-generation communication and computer technologies. In the last few decades, increasing attention has been paid to TE technology because of its ability to harvest and convert waste heat into electricity, leading to the efficient use of energy. Furthermore, miniaturized TE power generators can be used in portable and/or self-powered energy sources [1], including wearable electronics [2] and the Internet of things (IoT) systems [3].

The performance of a TE material is characterized by the dimensionless figure of merit *ZT* = *S*^2^*σT*/*κ*, where *S* is the Seebeck coefficient, *T* is the absolute temperature, *σ* is the electrical conductivity and *κ* is the thermal conductivity.

Semiconductors possess a greater TE effect than metallic materials, which led to the discovery of the first viable TE material, Bi_2_Te_3_. At the end of the last century, TE technology and materials received significant attention because of energy and environmental issues, and great progress has been made in both fundamental research and materials development. As the first TE material discovered ∼70 years ago, Bi_2_Te_3_-based materials remain at the forefront of TE research [4]; so far no TE materials have outperformed Bi_2_Te_3_ near room temperature.

Industrially, Bi_2_Te_3_-based TE materials (*ZT* < 1.0) are fabricated by zone melting and unidirectional solidification. The last decade has witnessed the development of Bi_2_Te_3_-based materials with even higher *ZT*. Figure 1 summarizes peak *ZT* (> 1) of Bi_2_Te_3_-based materials along with the years and optimal temperature regions in both *p*- and *n*-type materials [5,6]. All related references in Fig. 1 are listed in the Supplementary data (Table S1). Two trends were observed: *p*-type materials have higher *ZT*, while the peak *ZT* of *n*-type materials was obtained at higher temperatures. Although the same polycrystalline fabrication process was applied to *n*-type materials, expected performance enhancement has not been realized as in the *p*-type materials [7]. Nanocrystals allowed the peak *ZT* to reach up to 1.4 through intense phonon scattering by grain boundaries and defects [8]. Dispersed nano-SiC partially decoupled *S* and *σ* via an energy-filtering effect, leading to peak *ZT* as high as 1.33 at 373 K [9]. In 2015, an exceptional peak *ZT* of 1.86 was achieved by introducing dense dislocation arrays based on liquid-phase Te extrusion [10]. The idea of introducing dense dislocations has drawn great interest from researchers, leading to the recent studies devoted to processing innovations [11,12].

**Figure 1.**
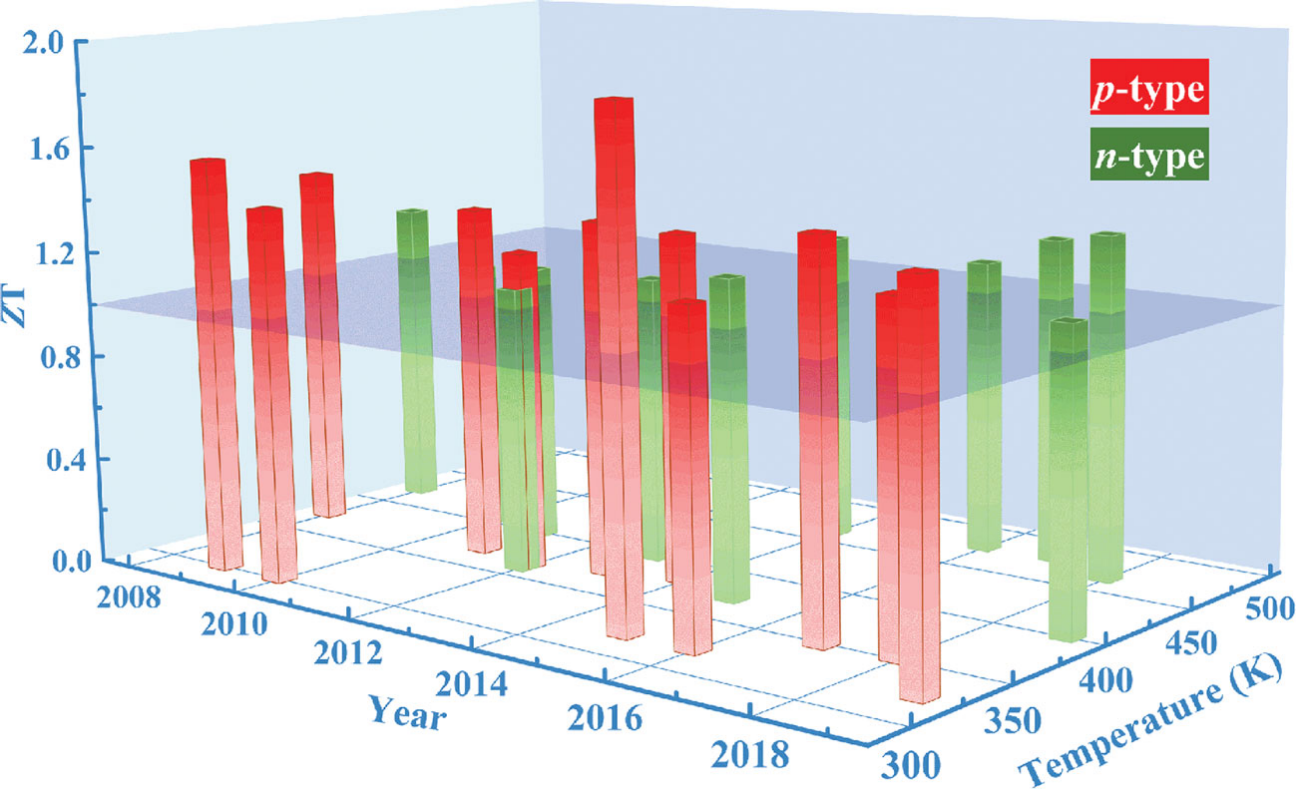
Collections of high peak *ZT* (>1) in Bi_2_Te_3_-based materials.

The peak *ZT* of *n*-type materials is often located at 400–470 K, which is higher than room temperature (Fig. 1). Commercial Bi_2_Te_3_-based materials are mainly used for refrigeration and cooling, and the optimal temperature region of *p*-type materials is near room temperature, making them more suitable for cooling applications. It is necessary to lower the optimal temperature region of *n*-type materials to near room temperature to match *p*-type materials and improve the overall performance of Bi_2_Te_3_-based TE devices.

Bi_2_Te_3_ has a rhombohedral structure with a quintuple-layer (QL) structure stacked in the order Te1-Bi-Te2-Bi-Te1 (Fig. 2a). The five atoms are bonded via mixed ionic-covalent bonding, and the interlayers are connected via van der Waals interactions. The calculated band structure of Bi_2_Te_3_ indicated an indirect band gap of 0.08 eV (Fig. 2b). Figure 2c shows the calculated electrical transport properties along the in-plane and out-of-plane directions, respectively. *μ*−*μ*_0_ represents the carrier concentration, and the sign of *μ*−*μ*_0_ indicates the semiconductor type. Calculations indicated that *p*-type Bi_2_Te_3_ materials possess higher *PF*/*τ* (*τ*: relaxation time) than *n*-type materials in both the in-plane and out-of-plane directions, because of higher band convergence and smaller band effective mass in valence band maxima. Detailed analysis can be found in the Supplementary data (Fig. S1). In addition, *PF*/*τ* is insensitive to *μ*−*μ*_0_ along the in-plane direction in *n*-type, implying that *PF* is insensitive to carrier concentration. Lattice thermal conductivity is similar in both *n*-type and *p*-type Bi_2_Te_3_ crystals [13]. The *PF*/*τ* difference between in-plane and out-of-plane directions for *n*-type materials is larger than for *p*-type materials, indicating that anisotropy more strongly affects the *ZT* in *n*-type Bi_2_Te_3_. This is why *n*-type polycrystalline materials always show inferior performance under the same fabrication processes.

**Figure 2.**
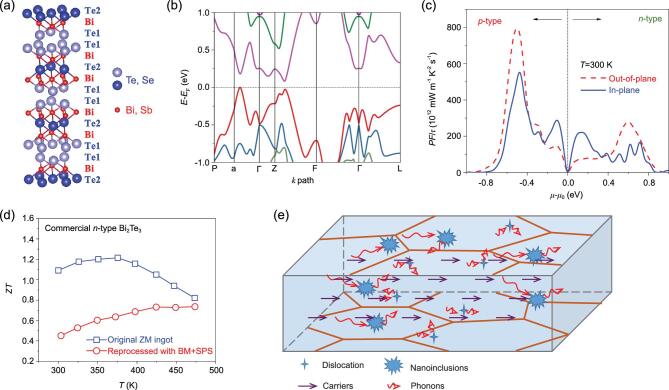
Fundamental features and research progress of Bi_2_Te_3_-based TE materials (a–c) showing the crystal structure, band structure and calculated power factor of Bi_2_Te_3_, respectively. (d) Comparison of *ZT*s of commercial *n*-type Bi_2_Te_3_-based ZM ingots and reprocessed bulks by BM + SPS. (e) Strategy for obtaining high-performance *n*-type Bi_2_Te_3_-based materials.

Conventional Bi_2_Te_3_-based ingots fabricated by zone melting (ZM) display preferred crystallographic orientations, leading to strong anisotropic electrical properties. The nanostructures formed by advanced sintering or deformation processes improve the performance of *p*-type materials (Fig. 1). Sintered materials usually show a lower electrical conductivity because of randomly oriented refined grains, but they also show reduced thermal conductivity because of enhanced phonon scattering resulting from microstructure refinement. However, as shown in Fig. 2d, the *ZT* of *n*-type materials decreased over a wide range when ingots fabricated by ZM were reprocessed into polycrystalline sintered materials using ball-milling (BM) and spark plasma sintering (SPS), as a result of the disappearance of intrinsic anisotropy and the increased carrier concentration originating from the `the donor-like effect'. Detailed analysis can be found in the Supplementary data (Fig. S2).

A breakthrough in the performance enhancement of *n*-type Bi_2_Te_3_-based materials requires creation of ideal microstructures with enhanced thermal resistivity and electrical conductivity. Fabricating nanostructured polycrystalline bulk materials appears to be a common method for *p*-type Bi_2_Te_3_ materials; however, fabricating polycrystals seems less suitable for *n*-type materials. Pan and Li [14] fabricated highly textured *n*-type Bi_2_(Te, Se)_3_ alloys with a further optimized composition by a repeated SPS hot-deformation process, which produced excellent carrier transport channels along the in-plane direction. Other works have shown similar results [15]. As shown in Fig. 2e, combining texturing with nanostructuring provides a balance between TE and mechanical performance in *n*-type Bi_2_Te_3_-based materials. Low/mid-frequency phonons are most likely to be scattered by nanoscale defects, thus significantly reducing the lattice thermal conductivity.

High efficiency TE devices are needed to meet the increasing demand for electronic cooling applications, which is driving current research activities devoted to performance enhancement for the *n*-type materials. Creating textured microstructure in favor of carrier transport, together with optimized nanostructure strengthening phonon scattering, could be an effective strategy. Additionally, the *n*-type base composition could be further tuned to raise the *ZT* values around room temperature, not by increasing the peak temperature.

## Supplementary Material

nwaa259_Supplemental_File
