# Supplementary material for: Bi2Te3-based applied thermoelectric materials: research advances and new challenges
Source: Natl Sci Rev. 2020 Oct 17;7(12):1856–8. doi: 10.1093/nsr/nwaa259 (PMC8290941; doi:10.1093/nsr/nwaa259)
Supplement: nwaa259_Supplemental_File [file nwaa259_supplemental_file.docx]

**Bi_2_Te_3_-based applied thermoelectric materials: research advances and new challenges**

Jun Pei#, Bowen Cai#*, Hua-Lu Zhuang, Jing-Feng Li*

State Key Laboratory of New Ceramics and Fine Processing, School of Materials Science and Engineering, Tsinghua University, 100084, Beijing, P. R. China.

Corresponding Author: [jingfeng@mail.tsinghua.edu.cn](mailto:majingfeng@mail.tsinghua.edu.cn); easoncc@mail.tsinghua.edu.cn

#contributed equally

**Table S1** The list of high performance *n*-type and *p*-type Bi_2_Te_3_-based TE materials

| Type | Composition | Method | *ZT*@Temperature | Year | Reference |
| --- | --- | --- | --- | --- | --- |
| *n*-type | Bi_2_Te_3_ | MA+HD | 1.19@420K | 2008 | [1] |
|  | Bi_2_Te_2.7_Se_0.3_ | MA+HD | 1.04@399K | 2010 | [2] |
|  | Cu_0.01_Bi_2_Te_2.7_Se_0.3_ | MA+HD | 1.06@398K | 2011 | [3] |
|  | Bi_2_Te_2.85_Se_0.15_+0.08 wt% I | ZM | 1.07@360K | 2012 | [4] |
|  | Cu_0.005_Bi_2_Te_2.7_Se_0.3_ | MA+HP | 1.08@398K | 2013 | [5] |
|  | Bi_2_Te_2.3_Se_0.7_ | Melting+BM+HD | 1.17@462K | 2014 | [6] |
|  | Bi_2_Te_2.79_Se_0.21_ | ZM+HD | 1.18@372K | 2015 | [7] |
|  | Bi_2_Te_2.2_Se_0.8_ | MA+HD | 1.11@475K | 2016 | [8] |
|  | Bi_2_Te_2.3_Se_0.69_ | LSM | 1.22@477K | 2017 | [9] |
|  | Bi_1.95_Sb_0.05_Te_2.3_Se_0.7_ | MA+HD | 1.28@465K | 2018 | [10] |
|  | Bi_2_Te_2.7_Se_0.3_+16 wt% Te | LPHD | 1.1@400K | 2019 | [11] |
| *p*-type | Bi_x_Sb_2-x_Te_3_ | MA+HP | 1.4@373K | 2008 | [12] |
|  | Bi_0.52_Sb_1.48_Te_3_ | MS+SPS | 1.56@300K | 2009 | [13] |
|  | Bi_0.5_Sb_1.5_Te_3_ | HD | 1.4@300K | 2010 | [14] |
|  | BiSbTe+0.75 wt% ZnAlO | Melting | 1.33@370K | 2011 | [15] |
|  | Bi_0.5_Sb_1.5_Te_3_ | Microwave solvothermal Method | 1.2@363K | 2012 | [16] |
|  | Bi_0.3_Sb_1.7_Te_3_+0.4 vol% SiC | MA+SPS | 1.33@373K | 2013 | [17] |
|  | Bi_0.3_Sb_1.7_Te_3_ | HD | 1.3@380K | 2014 | [18] |
|  | Bi_0.5_Sb_1.5_Te_3_+Te | Liquid-phase compaction | 1.86@320K | 2015 | [19] |
|  | Bi_0.5_Sb_1.5_Te_3_ | Solvothermal Method | 1.2@320K | 2016 | [20] |
|  | Bi_0.5_Sb_1.5_Te_3_ | Melting spinning | 1.42@348K | 2017 | [21] |
|  | Bi_0.5_Sb_1.5_Te_3_+20 wt% Te | Melt spinning+SPS | 1.24@350K | 2018 | [22] |
|  | Bi_0.4_Sb_1.6_Te_3_+0.2 at% Te | Two-step sintering process | 1.38@323K | 2019 | [23] |


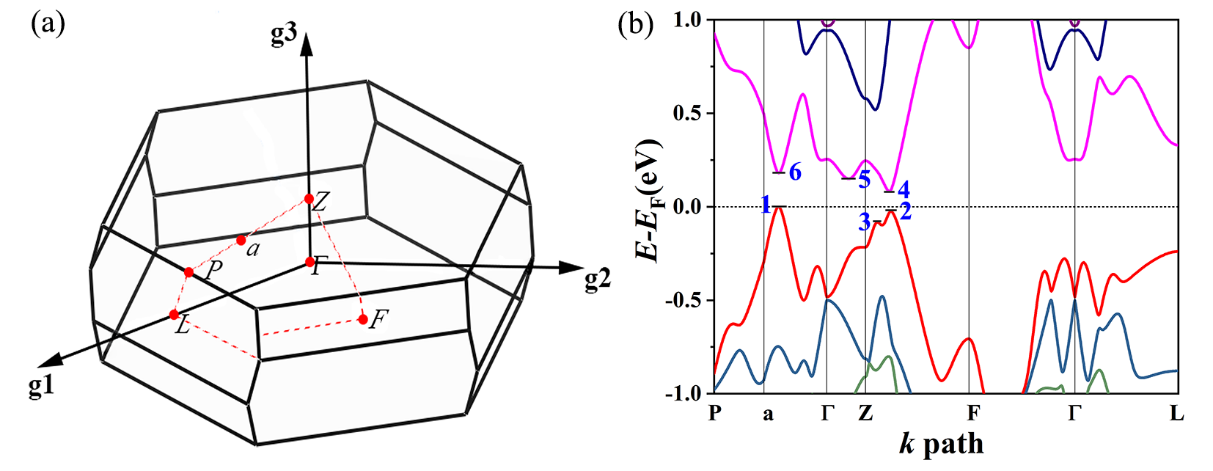


**Figure S1** Brillouin zone (a) and band structure (b) of Bi_2_Te_3_ crystal.

Figure S1 shows the Brillouin zone and band structure of Bi_2_Te_3_ crystal. The band structure is calculated along the P→a→Γ→Z→F→Γ→L path within first brillouin zone. The derived band gap of 0.08 eV is lower than that obtained from experiments (0.13-0.16 eV), which is ascribed to the underestimated band gap by adopting GGA-PBE function [24]. Owing to the spin-orbit coupling effect, multi-valley behavior can be observed in valence band and conduction band. Three valence bands lie close to each other in the energy range from ~-0.08 to 0 eV, which can be marked as 1, 2, 3, respectively. The energy offsets for 1-2, 1-3 are 0.024 eV and 0.08 eV, respectively. And three conduction bands locate close in the energy range from ~0.08 to 0.18 eV, which can be marked as 4, 5, 6, respectively. The energy offset of valence bands is larger than that of conduction bands, indicating higher convergence. Besides, the valence bands are sharper than that of conduction band, being indicative of smaller band effective mass. Owing to higher band convergence and smaller band effective mass, *p*-type Bi_2_Te_3_ would exhibit superior electrical performance.


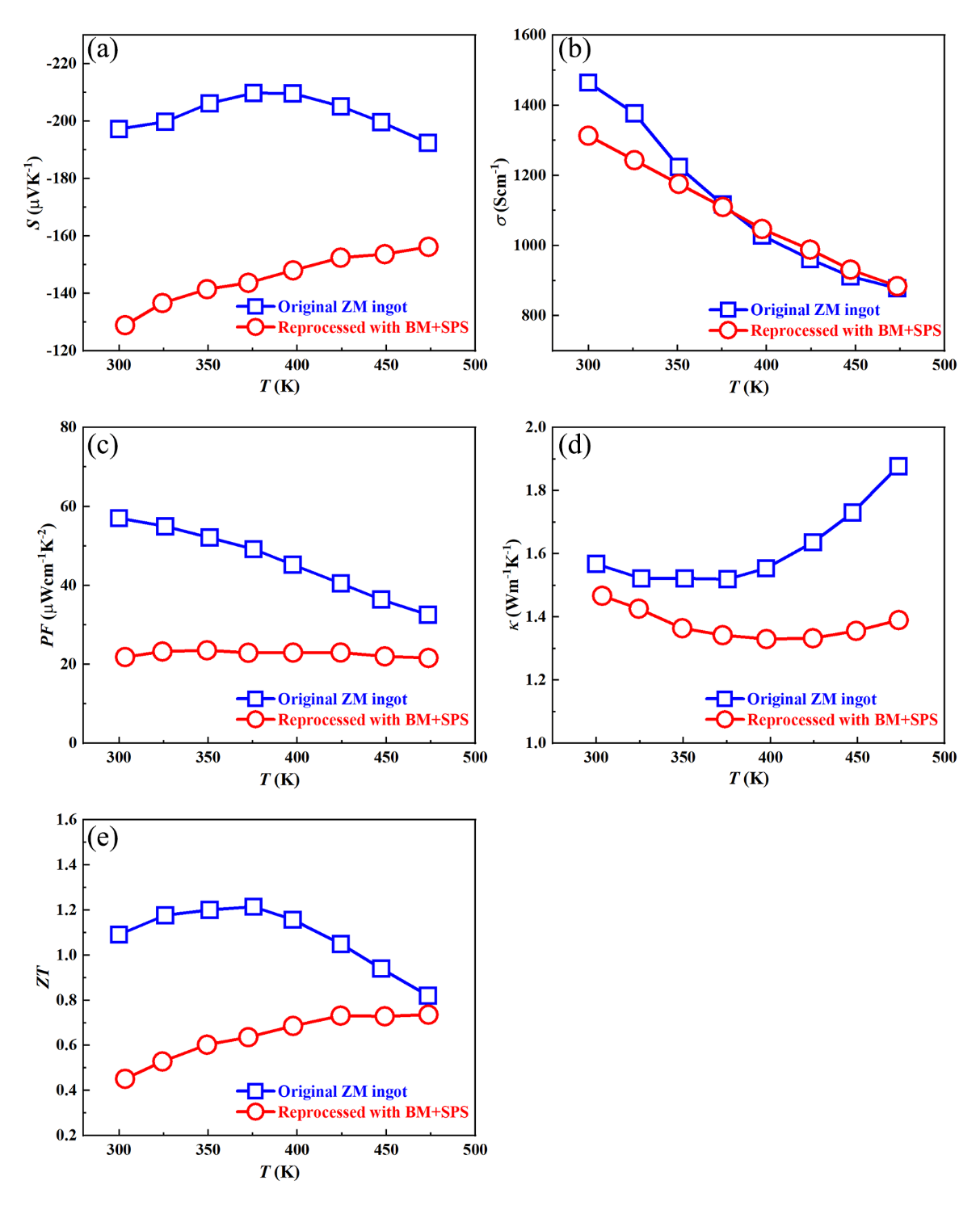


**Figure S2** Temperature dependence of TE transport for ZM ingot and reprocessed polycrystalline bulk sample. (a) Seebeck coefficient; (b) electrical conductivity; (c) power factor; (d) thermal conductivity; (e) *ZT*.

Figure S2 shows the temperature dependence of TE transport properties for ZM ingot and reprocessed polycrystalline bulk sample. *S* of the reprocessed polycrystalline bulk sample is distinct lower than that of ZM ingot, which can be attribute to the disappearance of intrinsic anisotropy and the increased carrier concentration originated from “donor-like” effect. Owing to the simultaneous reduction of *σ* and *S*, the *PF* of the reprocessed polycrystalline bulk sample is only half of that of ZM ingots. Figure S2d indications that the thermal conductivity (*κ*) of the reprocessed polycrystalline bulk sample is lower than that of ZM ingots. At higher temperature (>400 K), *κ* for ZM ingots increased rapidly with increasing temperature, while κ for the reprocessed polycrystalline bulk sample has no significant increasing. It can be attributed to the suppression of bipolar thermal conductivity resulted from the increasing carrier concentration. Therefore, the *ZT* of *n*-type materials decreased over a wide range when ingots fabricated by ZM were reprocessed into polycrystalline sintered materials using ball-milling (BM) and spark plasma sintering (SPS).

**References**

1. Zhao LD, Zhang B-P, Li J-F *et al.* Enhanced thermoelectric and mechanical properties in textured *n*-type Bi_2_Te_3_ prepared by spark plasma sintering. *Solid State Sci* 2008;**10**:651–8.

2. Yan X, Poudel B, Ma Y *et al.* Experimental studies on anisotropic thermoelectric properties and structures of *n*-type Bi _2_Te_2.7_ Se_0.3_. *Nano Lett* 2010;**10**:3373–8.

3. Liu W-S, Zhang Q, Lan Y *et al.* Thermoelectric property studies on Cu-doped *n*-type Cu_x_Bi_2_Te_2.7_Se_0.3_ nanocomposites. *Adv Energy Mater* 2011;**1**:577–87.

4. Wang S, Tan G, Xie W *et al.* Enhanced thermoelectric properties of Bi_2_(Te_1−x_Se_x_)_3_-based compounds as *n*-type legs for low-temperature power generation. *J Mater Chem* 2012;**22**:20943.

5. Liu W, Lukas KC, McEnaney K *et al.* Studies on the Bi_2_Te_3_–Bi_2_Se_3_–Bi_2_S_3_ system for mid-temperature thermoelectric energy conversion. *Energy Env Sci* 2013;**6**:552–60.

6. Hu L, Zhu T, Liu X *et al.* Point defect engineering of high-performance Bismuth-Telluride-based thermoelectric materials. *Adv Funct Mater* 2014;**24**:5211–8.

7. Hu L, Wu H, Zhu T *et al.* Tuning multiscale microstructures to enhance thermoelectric performance of *n*-type Bismuth-Telluride-based solid solutions. *Adv Energy Mater* 2015;**5**:1500411.

8. Pan Y, Li J-F. Thermoelectric performance enhancement in *n*-type Bi_2_(TeSe)_3_ alloys owing to nanoscale inhomogeneity combined with a spark plasma-textured microstructure. *NPG Asia Mater* 2016;**8**:e275–e275.

9. Zhu B, Huang Z-Y, Wang X-Y *et al.* Attaining ultrahigh thermoelectric performance of direction-solidified bulk *n*-type Bi_2_Te_2.4_Se_0.6_ via its liquid state treatment. *Nano Energy* 2017;**42**:8–16.

10. Hu L, Zhang Y, Wu H *et al.* Synergistic compositional-mechanical-thermal effects leading to a record high *ZT* in *n*-type V_2_VI_3_ alloys through progressive hot deformation. *Adv Funct Mater* 2018;**28**:1803617.

11. Wu Y, Yu Y, Zhang Q *et al.* Liquid‐phase hot deformation to enhance thermoelectric performance of *n*‐type Bismuth‐Telluride‐based solid solutions. *Adv Sci* 2019;**6**:1901702.

12. Poudel B, Hao Q, Ma Y *et al.* High-thermoelectric performance of nanostructured Bismuth Antimony Telluride bulk alloys. *Science* 2008;**320**:634–8.

13. Xie W, Tang X, Yan Y *et al.* Unique nanostructures and enhanced thermoelectric performance of melt-spun BiSbTe alloys. *Appl Phys Lett* 2009;**94**:102111.

14. Shen J-J, Zhu T-J, Zhao X-B *et al.* Recrystallization induced in situ nanostructures in bulk Bismuth Antimony Tellurides: a simple top down route and improved thermoelectric properties. *Energy Environ Sci* 2010;**3**:1519.

15. Zhang T, Zhang Q, Jiang J *et al.* Enhanced thermoelectric performance in *p*-type BiSbTe bulk alloy with nanoinclusion of ZnAlO. *Appl Phys Lett* 2011;**98**:022104.

16. Mehta RJ, Zhang Y, Karthik C *et al.* A new class of doped nanobulk high-figure-of-merit thermoelectrics by scalable bottom-up assembly. *Nat Mater* 2012;**11**:233–40.

17. Li J, Tan Q, Li J-F *et al.* BiSbTe-based nanocomposites with high *ZT*: the effect of SiC nanodispersion on thermoelectric properties. *Adv Funct Mater* 2013;**23**:4317–23.

18. Hu L, Zhu T, Liu X *et al.* Point defect engineering of high‐performance Bismuth‐Telluride‐based thermoelectric materials. *Adv Funct Mater* 2014;**24**:5211–8.

19. Kim SI, Lee KH, Mun HA *et al.* Dense dislocation arrays embedded in grain boundaries for high-performance bulk thermoelectrics. *Science* 2015;**348**:109–14.

20. Hong M, Chen ZG, Yang L *et al.* Bi_x_Sb_2−x_Te_3_ nanoplates with enhanced thermoelectric performance due to sufficiently decoupled electronic transport properties and strong wide-frequency phonon scatterings. *Nano Energy* 2016;**20**:144–55.

21. Yu Y, He D-S, Zhang S *et al.* Simultaneous optimization of electrical and thermal transport properties of Bi_0.5_Sb_1.5_Te_3_ thermoelectric alloy by twin boundary engineering. *Nano Energy* 2017;**37**:203–13.

22. Deng R, Su X, Zheng Z *et al.* Thermal conductivity in Bi_0.5_Sb_1.5_Te_3+x_ and the role of dense dislocation arrays at grain boundaries. *Sci Adv* 2018;**4**:eaar5606.

23. Pan Y, Qiu Y, Witting I *et al.* Synergistic modulation of mobility and thermal conductivity in (Bi,Sb)_2_Te_3_ towards high thermoelectric performance. *Energy Environ Sci* 2019;**12**:624–30.

24. Tran F, Blaha P, Schwarz K. Band gap calculations with Becke-Johnson exchange potential. *J Phys-Condens Matter* 2007;**19**:196208.
